# Detection of Anti-Nucleocapsid Antibody in COVID-19 Patients in Bangladesh Is not Correlated with Previous Dengue Infection

**DOI:** 10.3390/pathogens10060637

**Published:** 2021-05-22

**Authors:** Simon D. Lytton, Mahmuda Yeasmin, Asish Kumar Ghosh, Md. Rakibul Hassan Bulbul, Md. Maruf Ahmed Molla, Martha Herr, Helmut Duchmann, Md. Mohiuddin Sharif, Tasnim Nafisa, Md. Robed Amin, Nur Hosen, Md. Tanvir Rahman, Sumaiya Islam, Alimul Islam, Abul Khair Mohammad Shamsuzzaman

**Affiliations:** 1 SeraDiaLogistics, 81545 Munich, Germany; 2 National Institute of Laboratory Medicine and Referral Center, Sher E-Bangla Nagar, Dhaka 1207, Bangladesh; shumi.yeasmin@gmail.com (M.Y.); maruf063@gmail.com (M.M.A.M.); nafisahf3@gmail.com (T.N.); nurhosen568@gmail.com (N.H.); zaman.tushar@gmail.com (A.K.M.S.); 3 Dhaka Medical College Hospital, Dhaka 1000, Bangladesh; asish127kumar@gmail.com (A.K.G.); mohiuddinsharif@pircc.org (M.M.S.); robedamin@yahoo.com (M.R.A.); 4 Institute for Developing Science and Health Initiatives, Mohakhali, Dhaka 1212, Bangladesh; rakib@ideshi.org; 5 NovaTec Immundiagnostica GmbH, 63128 Dietzenbach, Germany; m.herr@novatec-id.com (M.H.); h.duchmann@novatec-id.com (H.D.); 6 Department of Microbiology and Hygiene, Faculty of Veterinary Science, Bangladesh Agricultural University, Mymensingh 2202, Bangladesh; tanvirahman@bau.edu.bd (M.T.R.); alimul.vmh@bau.edu.bd (A.I.); 7 Bangladesh Medical College and Hospital, 14/A Dhanmondi, Dhaka 1209, Bangladesh; sumaiya.islam.bmc@gmail.com

**Keywords:** COVID-19, dengue fever, SARS-CoV-2, N-protein, DENV, IgG, IgM, IgA, ELISA, rT-PCR

## Abstract

Background: The assessment of antibody responses to severe acute respiratory syndrome coronavirus-2 is potentially confounded by exposures to flaviviruses. The aims of the present research were to determine whether anti-dengue antibodies affect the viral load and the detection of anti-coronavirus nucleocapsid (N)-protein antibodies in coronavirus infectious disease 2019 (COVID-19) in Bangladesh. Methods: Viral RNA was evaluated in swab specimens from 115 COVID-19 patients by real-time reverse transcription polymerase chain reaction (rT-PCR). The anti-N-protein antibodies, anti-dengue virus E-protein antibodies and the dengue non-structural protein-1 were determined in serum from 115 COVID-19 patients, 30 acute dengue fever pre-COVID-19 pandemic and nine normal controls by ELISA. Results: The concentrations of viral RNA in the nasopharyngeal; Ct median (95% CI); 22 (21.9–23.3) was significantly higher than viral RNA concentrations in oropharyngeal swabs; and 29 (27–30.5) *p* < 0.0001. Viral RNA concentrations were not correlated with-dengue IgG levels. The anti-nucleocapsid antibodies were IgA 27% positive and IgG 35% positive at days 1 to 8 post-onset of COVID-19 symptoms versus IgA 0% and IgG 0% in dengue patients, *p* < 0.0001. The levels of anti- nucleocapsid IgA or IgG versus the levels of anti-dengue IgM or IgG revealed no significant correlations. Conclusions: Viral RNA and anti-nucleocapsid antibodies were detected in COVID-19 patients from dengue-endemic regions of Bangladesh, independently of the dengue IgG levels.

## 1. Introduction

The severe acute respiratory syndrome coronavirus-2 (SARS-CoV-2) is the etiologic agent of the coronavirus disease 2019 (COVID-19), which was first reported in December 2019 in Wuhan, China. COVID-19 was declared to be a pandemic by the World Health Organization (WHO) on 11 March 2020 [1] and until now it is a global health and global economic crisis. The original SARS-CoV-2 strain and its variants have infected over 166 million people and have caused an estimated 3.5 million deaths worldwide [2]. Industrialized countries in Europe and North America, China, Russia, and India are battling against SARS-CoV-2 transmission by mandatory face coverings, closure of culinary, sport and entertainment industries, and restrictions on travel. To avert further economic loss resulting from COVID-19, vast investments have been made in routine diagnostic testing and vaccine development. Thus far, little attention and resources have been given to investigate the potential consequences of SARS-CoV-2 circulating in populations with endemic Flaviviruses, which comprise more than 25 percent of the world’s population [3]. 

The single-stranded (ss) RNA and enveloped dengue fever viruses (DENV) and related flaviviruses are different to the circulating SARS-CoV-2 virus with respect to their modes of transmission, viral tropism, and molecular determinants of post-entry viral replication [3,4]. Despite the differences in molecular structure and replication, the clinical features of dengue fever and symptomatic COVID-19 exhibit striking similarities, acute flu-like symptoms 4–5 days and 5–7 days, respectively, after viral exposure, and fever, headache, cough, breathing difficulty, muscle ache, nausea and fatigue, and the release of pro-inflammatory cytokines [5,6,7,8,9]. The course of dengue fever and COVID-19 disease is usually mild and self-limiting after supportive care [6]. In hospitalized intensive care unit (ICU) patients, the viral illness suffering from either disease often leads to life-threatening cardiovascular and organ complications [7,8,9]. 

Dengue outbreak in 2019 in Bangladesh, primarily from DENV-3 serotype, reported 82,000 cases and 67 deaths, which surpassed all previous records [10]. Scenario assessments in 2020 predicted the harsh impact of dengue infection and climate conditions on the strategic response to COVID-19 and management of clinical outcomes [11,12]. Surprisingly, the cumulative total of 1000 dengue fever cases in 2020 indicates a dramatic decline in hospitalized dengue virus infection from the previous year [13,14]. In the same period, the Bangladesh Institute for Epidemiology Disease Control and Research (IEDCR) reported 533,000 SARS-CoV-2 infections and over 8000 deaths from COVID-19, of which 70% were more than 50 years of age and were predominantly male [15,16]. 

The emergence of pathogenic coronaviruses in flavivirus endemic populations has created an urgent need for SARS-CoV-2 serological tests, which discriminate between the anti-virus antibodies and accurately assess the seroprevalence of dengue fever and COVID-19 [16,17]. Cross-reactivity between SARS-CoV-2 spike (S) protein and dengue envelope protein is indicated in pre-COVID-19 pandemic serum from flavivirus infection; 20% of 19 confirmed Brazilian Zika infection [18] and 22% of 70 dengue IgG positive travelers from Israel and Italy [19] show positive IgM and/or IgG reactivity in SARS-CoV-2 S RBD rapid tests and/or semi-quantitative ELISA. Molecular docking and computational algorithms on SARS-CoV-2 S-protein X-ray crystal structure and anti-dengue envelope (E) monoclonal antibodies (MAbs) predict significant surface interactions between the anti-DENV E MAbs and the S-protein receptor binding domain (RBD), including amino acids K417, Y449, Y453, Q493, G495, Q498, T500 and Y505, which mediate direct contact with the angiotensin-converting enzyme-2 (ACE-2) receptor [20]. These data raise the concern of false-positive anti-dengue antibody reactivity in COVID-19 disease and false-positive anti-SARS-CoV-2 antibody reactivity in dengue fever patients. Furthermore, the data have spurred speculations that pre-exposure to dengue virus may confer protection against COVID-19 severity or SARS-CoV-2 antibodies can mediate antibody-dependent enhancement or worsen the course of infection with dengue viruses [20,21].

The N-protein structure [22], unlike the S-protein RBD [19,20,23], does not show cross-reactivity with anti-dengue antibodies [24,25,26,27] and is not targeted by the current approved COVID-19 vaccines [28]. Thus, the utility of N-protein immunoassays, rather than S-protein immunoassays, should be considered to assess the exposure to SARS-CoV-2 infection and to estimate the incidence of COVID-19 disease in dengue endemic populations. 

Considering the above implications, this study aimed to detect the anti-SARS-CoV-2 N-protein antibodies in dengue endemic populations of Bangladesh and to determine whether previous dengue infection affects the levels of anti-SARS-CoV-2 N-protein IgA and IgG measured by Novatec enzyme-based immunoabsorbent assay (Novalisa). 

## 2. Materials and Methods

### 2.1. Study Design 

Longitudinal and cross-sectional assessments of SARS-CoV-2 viral RNA, anti-SARS-CoV-2-N-protein antibodies, and anti-dengue antibodies in patients suffering from COVID-19 disease were undertaken between May 2020 and November 2020. Additionally, the retrospective analyses of the anti-SARS-CoV-2-N protein antibodies and anti-dengue antibodies in dengue fever patients hospitalized were also conducted during the pre-COVID-19 pandemic period between April 2019 and July 2019 in Dhaka. 

### 2.2. Patients

Patients presenting COVID-19 symptoms in the period between May 2020 and November 2020 were enrolled after referral of their nasopharyngeal (NP) oropharyngeal (OP) specimens for confirmatory SARS-CoV-2 rT-PCR testing and after consent to serum donations; cohort 1, residents in the Dhaka area (n = 48) with a median age of 33 ranged between 10 and 72 years, which tested SARS-CoV-2 rT-PCR positive at the National Reference Laboratory-Dhaka, and cohort 2, residents in Narayanganj or Dhaka (n = 67) with a median age of 48 ranged between 4 and 72 years, which tested SARS-CoV-2 rT-PCR negative at the National Reference Laboratory-Dhaka. Dengue fever patients hospitalized at Dhaka Medical College (n = 30) with a median age of 32 ranged between 16 and 70 with residual sera samples collected in the period between April 2019 and July 2019 [7] and stored at −80 °C. The control subjects, cohort 4, were non-febrile healthy subjects (n = 9) with a median age of 29 ranged between 22 and 50 and were employed at the University Mymensingh or Dhaka Medical College and did not report a history of dengue or COVID-19, and consented to donate serum between October and November 2020. 

### 2.3. Specimen Sampling

The specimens, NP or OP, collected on the day of onset of COVID-19 symptoms or within 3 days after the onset of symptoms, were transported on flocked swabs in 800 µL of Sansure storage reagent X1002E sealed inside screw tight vials at 2–8 °C and delivered within 24 h to the National Institute of Laboratory Medicine and Referral Center. Whole blood was drawn in 7.5 mL gel clot vacutainer activator tubes Cat. No. 367987 (Becton Dickinson) and the separated serum was stored at −80 °C until thawed for ELISA measurements. Serial sampling of serum from the COVID-19 patients confirmed SARS-CoV-2 rT-PCR positive began on either the same day of the swab specimen or 1 or 2 days after. The serum at follow-up visits were in some cases on days 3 to 7, at days 17 to 28 days and at days 65 to 177 days after the onset of COVID-19 symptoms. Serum from all other patient groups were of a single time point; the SARS-CoV-2 rT-PCR negative on day 1 to 13 days after the onset of COVID-19 symptoms and the hospitalized dengue fever patients within 7 days after the date of the first reported fever.

### 2.4. Measurement of SARS-CoV-2 RNA

The SARS-CoV-2 RNA was extracted from a 10 µL of sample without heat inactivation using the direct 1-step Quick-Start Protocol 2019-nCoV kits (Sansure Biotech, Changsha, Hunan Province, China). The remaining sample material was stored at −80 °C. The QuantStudio 5/6 (Thermo Fisher, OMC Ltd, Dhaka, Bangladesh) and ABI 7500 fast (Thermo Fischer Applied Biosystems, OMC Ltd, Dhaka, Bangladesh) real time PCR were used to collect the fluorescent signals from the coronavirus open reading frame (ORF) on the FAM channel and the N-gene on the ROX channel. The CY5channel was selected to test the internal control. After adjusting the threshold values on each of the fluorophores for detection of the S-type amplification curves, the Ct values of <40 for either N gene or ORF region were reported as positive. 

### 2.5. Serological Testing

The dengue IgM and IgG, the dengue non-structural protein-1 (NS-1) and the SARS CoV-2 IgA and IgG were analyzed by specific ELISA in accordance with the manufacturer’s instructions and recommended cut-off value of NovaTec units (NTU) > 10 for positive results (Novatec Diagnostics, Dietzenbach, Germany); NTU = X *10/QC where X = OD_450nm_ − OD_620nm_ of the test sample and QC = OD_450nm_ − OD_620nm_ of the quality control equivocal serum sample. ELISA measurements were all performed on an ANTHOS READER HT1 plate reader.

### 2.6. Statistical Analysis

The IgM and IgG levels are presented as the mean with a standard deviation and medians with ranges. Comparisons of the values between patient groups were assessed by non-parametric Mann-Whitney sum rank test. A *p* value of ≤0.05 was considered to be statistically significant with differences between independent groups. Correlation analyses were calculated with the Spearman’s rank correlation coefficients. The correlation coefficients of r > 0.4 or r < −0.4 with significance at *p* < 0.05 were considered to be strongly positive or strong negative associations, respectively. Statistical analysis was done with MedCalc version 14 for Windows (MedCalc Software, Mariakerke, Belgium).

## 3. Results

### 3.1. Patient Groups and Clinical Data

**Figure 1:**
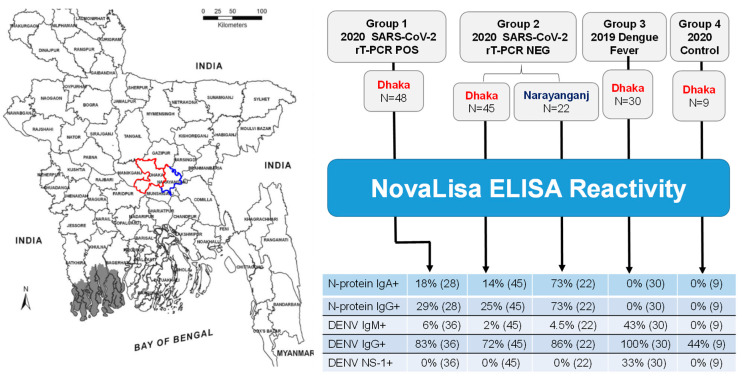
Study Plan. Elisa reactivity was assessed in four independent patient groups. Group 1; n = 48 COVID-19 patients in Dhaka were confirmed to be SARS-CoV-2 rT-PCR positive, Group 2; COVID-19 in two neighboring districts that tested negative SARS-CoV-2 by rT-PCR; Dhaka (n = 45) district boundary outlined in red and Nayaranganj (n = 22) district boundary outlined in blue. Group 3; Pre-pandemic 2019 Dengue patients and Group 4; Control subjects in Dhaka during the 2020 COVID-19 pandemic. District boundaries adapted from the map of Bangladesh [29].

In this study, the demographic (age and gender) and clinical data (COVID-19 and dengue symptoms) of 154 enrolled patients was evaluated; 115 COVID-19, of which 48 were rT-PCR confirmed positive (group 1) and 67 were SARS-CoV-2 rT-PCR negative (group 2), 30 pre-pandemic hospitalized dengue fever (group 3) and nine healthy adult controls (group 4) who had no known exposure to SARS-CoV-2 and were without dengue infection during the 2020 post-pandemic period (Figure 1, Table 1). The gender bias, which had two–three fold higher frequency of males than females, was found across all four patient groups. The median age of group 1 (48 years) was higher than the median age of the other groups (29–33 years). SARS CoV-2 rT-PCR testing was performed on nasal or nasopharyngeal specimens taken from patients in the two COVID-19 cohorts with a median of two days after the onset of symptoms (Table 1). COVID-19 symptoms in both the rT-PCR positive cohort and rT-PCR negative cohort were mostly mild or moderate. The six COVID-19 patients reporting severe illness were confirmed as SARS-CoV-2 rT-PCR positive and received supplementary oxygen in accordance with the WHO guidelines. The pre-pandemic dengue patients were all hospitalized within seven days of the first day of fever and showed significant thrombocytopenia (Table 1). Seven to twenty percent of the dengue patients presented one or more warning signs. 

### 3.2. SARS-CoV-2 RNA Levels

**Figure 2:**
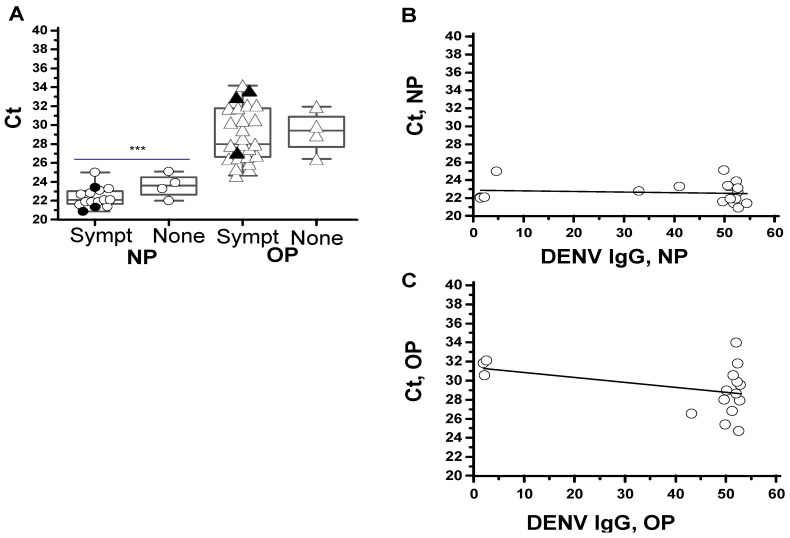
SARS-CoV-2 RNA levels and correlations with dengue IgG levels. The boxplot corresponds to the 25th and 75th interquartile range (IQR), the horizontal line inside the box to the median and the bars outside the box to the minimum and maximum RNA concentrations (Ct values). The Ct values in n = 19 nasopharyngeal specimens (NP, circles) with a mean of 22.6 (95% CI 22–23) versus n = 29 oropharyngeal specimens (OP, triangles) median of 29 (95% CI 28–30) *** *p* < 0.0001. The Ct values of NP specimens from COVID-19 patients reporting symptoms (sympt) with a mean of 22 (95% CI 21.8–23) versus no symptoms (none) with a mean of 23.6 (21.5–25.6) *p* = 0.06. The filled symbols represent severe cases (**A**). The scatter plots represented by open circles and the curve fit of linear regression analysis of Ct values and anti-dengue (DENV) IgG levels are represented by the solid line (**B** and **C**); n = 19 NP specimens and matched serum; r = −0.11 (95% CI −0.54–0.36), *p* = 0.65 (**B**) and n = 17 OP specimens and matched serum; r = 0.38 (95% CI −0.73–0.12, *p* = 0.3.

The 48 patients confirmed to be SARS-CoV-2 rT-PCR positive at days one to eight after the onset of COVID-19 symptoms had viral RNA extracted from NP or OP swabs. The NP sampling gave significantly higher viral RNA concentrations than the OP sampling (Figure 2A). The trend of higher RNA levels (lower Ct values) in the COVID-19 patients with symptoms versus without symptoms did not reach significance. Four of the six severe COVID-19 patients reported Ct values below the median Ct of COVID-19 patients without symptoms (Figure 2A). In 36 COVID-19 patients, the serum matched the date of the swab specimens, which were investigated for anti-dengue antibodies. No correlations of significance were found between the Ct values of either NP (Figure 2B) or OP (Figure 2C) specimens with the anti-dengue IgG levels.

### 3.3. Temporal Profile of Anti-SARS-CoV-2 N-Protein Antibodies

**Figure 3:**
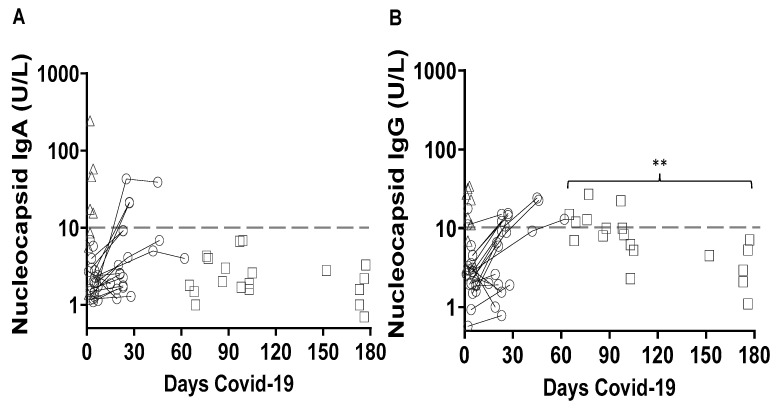
Kinetics of anti-SARS-CoV-2 N-protein antibodies in rT-PCR positive COVID-19. The IgA (**A**) and IgG (**B**) levels in the course of n = 48 COVID-19 patients. The horizontal dotted line represents the 10 U/L cut-off for positive reactivity in ELISA. Serial measurements of n = 17 patients (open circles) and single time point measurements of n = 12 patients between day 1 to day 8 (open triangles) or n = 19 patients between day 65 to day 177 (open squares). ** *p* = 0.001 paired student *t*-test comparing the mean IgA level and the mean IgG level at interval day 65 to day 177.

To assess the antibody responses to SARS-CoV-2 N-protein, the IgA and IgG levels were compared at one to eight days post-onset of COVID-19 symptoms and then at two or three intervals in the follow-up visits of the SARS-CoV-2 rT-PCR positive COVID-19 patients (Figure 3). The anti-N-protein IgA was detected in 18, 31 and 33 percent of the patients on days one to eight, on days 15 to 28 and on days 42 to 62, respectively (Table 2, Figure 3A). After 62 days, all anti-N-protein IgA values were below the cut-off (Figure 3A). The anti-N-protein IgG remained above the cut-off in 37 percent of the SARS-CoV-2 rT-PCR patients at day 65 to day 177 after the onset of COVID-19 symptoms with anti-N-protein IgG levels significantly higher than the levels of anti-N-protein IgA during this period (Figure 3B).

### 3.4. Anti-SARS-CoV-2 N Protein and Anti-Dengue E Protein Antibodies in COVID-19 Patients and in Pre-Pandemic COVID-19 Dengue Patients

The percentage of patients with positive anti-N-protein antibodies on day one to eight post-onset of COVID-19 symptoms was higher in cohort 2 than in cohort 1 (Table 2). No clinical signs of acute dengue infection and no serum NS-1 positivity was detected in the two COVID-19 cohorts. The percentage of patients in cohort 1 showing positive anti-dengue IgM and IgG was 6 and 81 percent, respectively, versus, 3 and 83 percent, respectively, in cohort 2 (Table 2). The data confirm the high dengue IgG seroprevalence known for the dengue endemic populations of Bangladesh [10,13]. 

**Figure 4:**
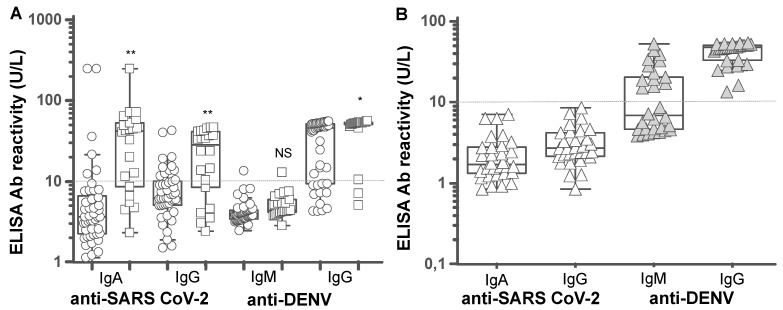
Levels of anti-SARS-CoV-2 N-protein antibodies and anti-dengue antibodies. The N-protein IgA and IgG and the dengue IgM and IgG in rT-PCR negative COVID-19 (**A**) and in pre-pandemic hospitalized dengue fever (**B**). The difference in mean antibody activities between the two districts were compared by independent student t-tests. In Dhaka city, n = 45 (circles) anti-N-protein specific antibody levels IgA; mean 16 [95% CI 0.8–32], 14% positive and IgG; mean 9 (95% CI 6.7–11.8), 25% positive versus Narayanganj district n = 22 (squares), IgA; mean activity 43 (95% CI 20–66), 73% positive ** *p* < 0.001 and IgG; mean activity 25 (95% CI 18–32), 73% positive ** *p* < 0.001. The anti-dengue IgM in Dhaka city mean activity 4.3 (95% CI 3.8–4.9) 2.3%positive versus Narayanganj district; mean activity 5.3 (95% CI 4.4–6.2) 4.5% positive, *p* = 0.07, and anti-dengue IgG; mean activity 34 [95% CI 28–40] 72% positive versus mean activity 46 (95% CI 39–53), 86% positive, respectively, * *p* = 0.01. NS = not significant.

In order to determine whether the seroprevalence of anti-SARS-CoV-2 antibodies differs between districts in Bangladesh reporting the country’s highest infection rates of 30, 31 the patients in cohort 2 were sub-divided into two groups; 45 cases from Dhaka city and 22 cases in the adjacent Narayanganj district located 30 km South East, where the first COVID-19 cases were reported in March 2020 (Figure 1). Both Dhaka city and Narayanganj district are COVID-19 hotspots [30,31,32]. The percentage positivity and the levels of anti-N-protein antibody in Narayanganj was significantly higher than that found in Dhaka city; anti-N-protein specific IgA positivity was 73% versus 14% with 10–20-fold higher levels and the anti-N-protein specific IgG positivity of 73% versus 25% with two–five-fold higher levels (Figure 4A). In the SARS-CoV-2 rT-PCR positive COVID-19, all were from Dhaka city; 18% were positive for IgA; and 29% were positive for IgG at one–eight days (Table 2), which was similar to the percentages of rT-PCR negative COVID-19 in Dhaka city (Figure 4A).

Positive anti-dengue IgM was detected in two cases, one from each district (Figure 4A). Anti-dengue IgG positivity was found in 72 and 86 percent of the cohort 2 COVID-19 cases from Dhaka city and Naravanganj district, respectively, and showed no significant differences from the 83 percent positive anti-dengue IgG found in cohort 1. In the thirty pre-COVID-19 dengue fever patients, anti-dengue IgM positivity of 43 percent and IgG positivity of 100 percent was detected (Figure 4B).

### 3.5. Correlations of Serum SARS-CoV-2 N-Protein IgG and IgA Levels to Dengue E Protein IgG Levels

**Figure 5:**
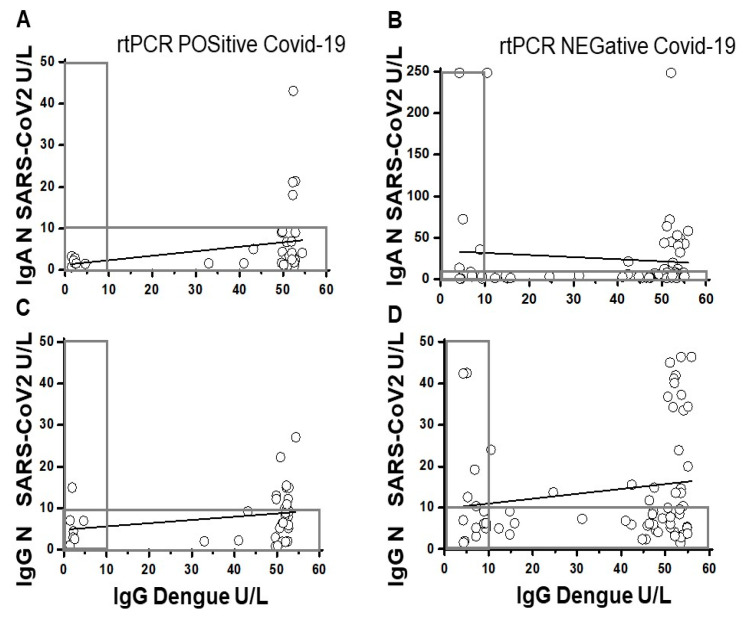
Correlations of SARS CoV-2 N protein IgA or IgG and Dengue IgG. The anti-N-protein IgA (**A**) and (**B**) and IgG (**C**) and (**D**) at 14–90 days after COVID-19 symptoms in n = 36 rT−PCR positive (**A**) and (**C**) and at 1–8 days after COVID-19 symptoms in n = 67 rT-PCR negative patients (**B**,**D**). The values within the area of the box are below the 10 U/L cut-off for positive reactivity in ELISA. The coefficient of correlations between dengue IgG and N-protein IgA in rT-PCR pos COVID-19; r = 0.26 95% CI −0.07–0.54), *p* = 0.127 and in rT-PCR neg COVID-19; r = −0.28 (95% CI −0.53–0.03), *p* = 0.07. The coefficient of correlations between dengue IgG and N-protein IgG in rT-PCR pos COVID-19; 0.44 (95% CI 0.03–0.73), *p* = 0.04 and in rT-PCR neg COVID-19; r = −0.33 (95% CI −0.64–0.08), *p* = 0.11.

To investigate whether the antibody responses to SARS-CoV-2 N-protein are affected by previous exposure to dengue viruses, the levels of N-protein specific IgA and IgG versus the levels of anti-DENV E protein IgG in COVID-19 patients were assessed using linear regression analyses (Figure 5). In both COVID-19 cohorts, no correlations of significance were found between the anti-N-protein specific IgA (Figure 5A,B) or IgG (Figure 5C,D), and the anti-dengue E protein IgG. 

## 4. Discussion

Anti-SARS-CoV-2 antibodies measured in different countries throughout the COVID-19 pandemic reveal three distinguishing features: first, the titers of anti-S-protein and anti-N-protein antibodies among severe cases in ICU or hospitalization are higher than titers among mild or moderate COVID-19 disease [33,34,35,36]. The percentage of positivity of anti-S-protein antibodies are greater than the percent positivity of anti-N-protein antibodies measured on the Luminex trimeric S protein immunoassay within the first 30 days post-onset of COVID-19 symptoms [37]. Second, greater than 90 percent of the COVID-19 patients show anti-S-protein antibody positivity within ninety days post-onset of COVID-19 symptoms [38,39,40,41]. Anti S-protein antibodies in the S-protein IgG ELISA and in automated immunoassays strongly correlate with the activity of neutralizing antibodies detected by the surrogate cell-based viral inhibition assay [23,39,40,41,42]. The S-protein immunoassays reliably detect antibody responses to S-protein and S protein ACE-2 RBD in COVID-19 vaccination trials [43,44]. Third, the N-protein ELISA from different manufacturers show analytical sensitivities ranging from 82 to 99 percent and analytical specificities ranging from 98 to 100 percent for specific IgA and IgG on day 7 to day 14 after rT-PCR confirmed SARS-CoV-2 infection [45,46,47,48,49,50]. In some cases, the N-protein specific IgA levels increase by 10 to 100-fold above the immunoassay cut-off, indicating a rigorous mucosal immune response to SARS-CoV-2 [51]. The seroconversion from N-protein specific IgA (not IgM) to N-protein specific IgG within 30 days after the onset of COVID-19 symptoms and the IgG levels, which persist above the cut-off 90–120 days after the onset of COVID-19 symptoms, are the best metrics for the assessment of the seroprevalence and estimating the exposure to SARS-CoV-2 in populations [52,53,54,55].

A previous immune surveillance study on anti-SARS-CoV-2 S-protein antibody responses in 108 mild symptomatic and 63 asymptomatic COVID-19 disease in Dhaka Bangladesh between March and August 2020 found 72 and 83 percent positive anti-S protein specific IgA and IgM, respectively, on day one and day seven and 100 percent positive anti-S protein specific IgG on day 7, day 14 and day 30 after the date of confirmed rT-PCR testing [56]. No anti-S protein ELISA reactivity was detected in the pre-COVID-19 pandemic control group, including twenty Japanese encephalitis virus and fifty-nine confirmed influenza cases [56]. To the best of our knowledge, our study is the first report on antibody responses to the SARS CoV-2 N-protein in Bangladesh. In light of the previous reports on pre-pandemic flavivirus serum showing cross-reactivity with SARS-CoV-2 S protein [18,19,21] and the predictions of shared epitopes on S-protein RBD and dengue E protein [20], the question was asked whether the detection of N-protein specific IgA or IgG, unrelated to the neutralizing antibodies against the S-protein target, is affected by the anti-dengue IgG present in Bangladeshi patients suffering from COVID-19 disease and having previous dengue infection.

The N-protein specific antibodies were assessed in the COVID-19 patients that tested SARS-CoV-2 by rT-PCR positive and in the patients that tested SARS-CoV-2 by rTPCR negative within 1 week after the onset of COVID-19 symptoms. In the rT-PCR positive COVID-19 cohort, the SARS-CoV-2 RNA levels were higher in the NP specimens than the OP specimens. However the levels of SARS-CoV-2 RNA in COVID-19 patients was not correlated with anti-dengue IgG. These results underscore the greater sensitivity of rT-PCR in NP than in OP swabs and indicate that SARS-CoV-2 viral replication in the upper airway is not affected by the serum anti-dengue IgG levels.

The kinetics and magnitude of the SARS-CoV-2 antibody response was determined by comparing the percent positivity and the levels of anti-N-protein antibody at day one to day eight and at follow up intervals days 15 to 28, days 42 to 62 and days 65 to 177 post-onset of COVID-19 symptoms. The N-protein Novalisa uses glycosylated recombinant C-terminus of the SARS-CoV-2 N-protein, which, in a previous evaluation with 119 pre-pandemic control serum, including 14 serum with reactivity against MERS-CoV or seasonal coronaviruses, gave no false positive anti-N-protein IgG test results [47,48].

The Novatec units of antibody activity is established as a validated index for the semi-quantitative determination of anti-SARS-CoV-2 specific IgA [49,50] and IgG [48,49,50] at the manufacturer cut-off of 10 U/L. The analytical sensitivity 95% (95% CI 83–98%) and specificity 96% (95% CI 89–99%) for anti-N-protein IgG and the sensitivity of 90% (95% CI 76–96%) and specificity of 99% (95% CI 93–99%) for anti-N-protein IgA was previously determined on 208 Belgian patients with moderate to severe COVID-19 symptoms between days 14 to 18 after the onset of symptoms and 79 pre-COVID-19 pandemic serum representatives of various non-flavivirus and non-coronavirus infectious diseases [50]. In German health care workers with mild to moderate COVID-19 disease, the Novatec N-protein IgG ELISA gave a sensitivity of 72% (95% CI 51–88 %) at 14 to 21 days rT-PCR SARS-CoV-2 positivity, slightly lower than the 80% sensitivity reported by automated N-protein immunoassays of Siemens, Roche and Abbott [46]. The anti-N-protein IgG percent positives in Novalisa were 64, 87 and 88 percent at less than 7 days, 8 to 14 days and greater than 14 days post-onset of COVID-19 symptoms, respectively, in 101 Qatar patients with rT-PCR confirmed COVID-19 [48]. However, in Bangladesh, we cannot rule out exposures to coronaviruses pre-COVID-19 pandemic that may give rise to positive reactivity in the N-protein Novalisa.

The peak anti-N-protein IgA response occurred between 15 and 28 days post-onset of COVID-19 symptoms and no anti-N-protein IgA was detected greater than 65 days post-onset of COVID-19 symptoms. In contrast, positive anti-N protein IgG was found in 37 percent of the patients greater than 65 days post-onset of COVID-19 symptoms. These results are consistent with the anti-N-protein antibody kinetics previously determined by N-protein IgA and IgG Novalisa [46]. However, the 37 percent anti-N-protein IgG positivity is conspicuously lower than the 100 percent anti-N-protein Novalisa IgG previously reported at 56 days post-rT-PCR confirmed COVID-19 in Belgium [46] and the 90 percent anti-N-protein Novalisa IgG positivity greater than 30 days post-rT-PCR confirmed COVID-19 in Qatar [48]. 

On March 8, 2020, the first COVID-19 case in Bangladesh was reported. Between March 2020 and August 2020, the COVID-19 incidence in Dhaka City and Narayanganj districts was estimated at 151 and 73 SARS-CoV-2 by rT-PCR confirmed cases per 100,000 inhabitants, respectively [31,32,33]. These two districts have the highest population density in Bangladesh and were identified as the country’s epicentre. In September 2020, the government surveys on asymptomatic and mild symptomatic COVID-19 in Dhaka and neighboring districts reported discrepant findings of 10 percent and 90 percent of the COVID-19 cases as confirmed SARS-CoV-2 rT-PCR positive [56]. 

The percent positivity of anti-N-protein IgA and N-protein IgG was 43 and 73 percent, respectively, in the Narayanganj district; five-fold and three-fold higher, respectively, than the percent positivity of the anti-N-protein IgA and IgG in Dhaka City. The levels of N-protein IgA and IgG in the Narayanganj district were significantly higher than that of the IgA and IgG levels in Dhaka city. The findings are puzzling because these neighboring districts both reported mild to moderate COVID-19 disease and the serum sampling from adults was within 1 week after onset of COVID-19 symptoms. In the period between 1 October and 1 December 2020, we have not determined whether re-infection occurred with SARS-CoV-2 variants having viral genomes of lineages dissimilar to the first COVID-9 episode between March and August 2020 [57,58]. Nevertheless the anti-N-protein antibodies from Naravangaj within only one week after the onset of COVID-19 symptoms likely represent memory B-cell responses after re-infection with SARS-CoV-2, whereas the COVID-19 in Dhaka city indicate SARS-CoV-2 N-protein antibody responses in primary exposure. The COVID-19 disease among predominantly male adults is consistent with previous studies on the community spread of SARS-CoV-2 in the absence of social distancing and protective measures and male commuters who returned to work in Dhaka industries, despite nation-wide lockdown and the stay-at-home orders by the government of Bangladesh [59,60].

Of the thirty acute dengue infection patients from the 2019 dengue outbreak in Dhaka, none had positivity against the N-protein. This result is consistent with the previous evaluation of Novalisa, which reported 99% specificity of N-protein specific IgG using 79 serum from pre-COVID-19 pandemic [50] and the qualitative nucleoprotein-based Genescript ELISA, which reported 90 percent sensitivity for the detection of SARS-COV-2 IgG antibodies and no cross-reactivity in 76 serum of confirmed dengue and in 20 serum of confirmed Zika infections taken pre-COVID-19 pandemic from Brazilians [26]. We acknowledge that two of the 30 acute Bangladeshi dengue serum show borderline reactivity in the range 9 to10 U/L and may warrant expanded testing with a larger number of serum from the pre-COVID-19 pandemic dengue infections in order to confirm the specificity of the N-protein Novalisa.

No significant correlations were found by linear regression analyses of the N-protein specific IgG and IgA versus the anti-dengue IgG reactivity. These analyses included twenty-eight rT-PCR SARS-CoV-2 positive patients at 14 to 90 days post COVID-19 symptoms and sixty-seven rT-PCR SARS-CoV-2 negative patients at one-eight days post COVID-19 symptoms. The low numbers of follow up samples in the SARS-CoV-2 rT-PCR positive COVID-19 group and the unavailability of serum after eight days of COVID-19 symptoms in the SARS-CoV-2 rTPCR negative COVID-19 group are a limitation of our study. The data likely underestimate the positive anti-N-protein IgG reactivity, which develops after day eight. Despite these limitations attributed to logistical challenges of obtaining follow-up blood donations in Bangladesh patients after recovery from COVID-19, the results in this study underscore the reliability of the N-protein immunoassay for the detection of N-protein specific IgA and IgG during natural SARS-CoV-2 infection.

In dengue endemic populations of Asian and South American countries now infected with SARS-CoV-2 variants, the concern is over increased transmissiblity and the uncertainty of immune protection conferred by COVID-19 vaccines of different manufacturers and suppliers. The open question is whether the immune responses to S-protein are affected by pre-existing antibodies to regional flaviviruses that cross-react with S-protein RBD and/or exposures to SARS-CoV-2 variants that potentially escape immune recognition? For this reason, commercially approved S-protein immunoassays, which detect anti-S-protein or anti-S RBD antibodies, require similar evaluation of clinical performance as reported here for the N-protein Novalisa kit. Both the S-protein and N-protein immunoassays offer advantages in the overall assessment of anti-SARS-CoV-2 antibodies; the former detects neutralizing antibodies and the latter determines the seroprevalence of natural SARS-CoV-2 infection, independent of vaccination. The results in our study do not resolve all foreseeable questions relating to potential cross reactivity to SARS-CoV-2 N-protein in serum from flavivirus infection, but do add certainty that the detection of anti-N-protein antibodies by ELISA in patients suffering COVID-19 is not affected by prior exposure to dengue viruses.

## Figures and Tables

**Table 1:** Patient characteristics of COVID-19 and Dengue Fever in Bangladesh.

Characteristics	SARS-CoV-2 rtpcr POS Group 1	SARS-CoV-2 rtpcr NEG Group 2	Dengue 2019 Group 3	Bangladeshi Controls Group 4	*p*-Value (1)	*p*-Value (2)
**Number of cases enrolled**	N = 48	N = 67	N = 30	N = 9		
**Age Median (Range) years**	33 (10–72)	48 (4–72)	32 (16–70)	29 (22–50)	**0.001**	0.78
**Gender M/F**	35/13	41/26	23/7	8/1	0.197	0.46
**COVID-19**						
Severe n (%)	6 (12.5)	0 (0)	NA	NA	**0.003**	NA
Mild or Moderate n (%)	42 (87.5)	67 (100)	NA	NA	**0.003**	NA
Hospitalized n (%)	6 (12.5)	NA	NA	NA	NA	NA
Oxygen support n (%)	6 (12.5)	NA	NA	NA	NA	NA
Days fever mean (95% CI)	4.7 (4–5.3)	NA	NA	NA	NA	NA
Days after onset COVID-19 symptoms median (range)	2 (1–7)	2 (1–8)	NA	NA	0.9	NA
**COVID-19 symptoms n (%)**						
No symptoms	8 (17)	5 (7)	NA	NA	0.09	NA
Difficulty breathing	5 (10)	0 (0)	NA	NA	**0.008**	NA
Fever	31 (65)	52 (78)	NA	NA	0.13	NA
Cough	33 (69)	23 (34)	NA	NA	**0.0002**	NA
Malaise	5 (10)	4 (6)	NA	NA	0.43	NA
Muscle or body pain or headache	6 (13)	19 (28)	NA	NA	0.06	NA
Loss of smell and/ or taste	10 (21)	9 (13)	NA	NA	0.25	NA
Acute watery diarrhea	1 (2)	5 (7)	NA	NA	0.22	NA
**Dengue status**						
Primary infection %	NA	NA	0	NA	NA	NA
Secondary infection %	NA	NA	100	NA	NA	NA
Platelet count × 10^3^/µL Mean (95% CI)	NA	NA	50 (40–61)	NA	NA	NA
Days 1st Dengue fever Mean (95% CI)	NA	NA	4.2 (3–5)	NA	NA	NA
**Dengue symptoms n (%)**						
Fever	NA	NA	30 (100)	NA	NA	NA
Muscle or body pain or headache	NA	NA	15 (50)	NA	NA	NA
**Dengue warning signs n (%)**						
Plasma leakage	NA	NA	2 (7)	NA	NA	NA
Ascites	NA	NA	4 (13)	NA	NA	NA
Gum bleeding	NA	NA	6 (20)	NA	NA	NA
Nasal bleeding	NA	NA	3 (10)	NA	NA	NA
Nausea vomiting	NA	NA	5 (17)	NA	NA	NA

Normal ranges; Platelets 150–300 × 10^3^/µL. NA = not available or not applicable. NS = not significant. (1) Comparison between cohort 1 versus cohort 2; (2) between cohort 1 versus cohort 3.

**Table 2:** Antibody positivity in COVID-19 and Dengue Fever in Bangladesh.

Antibody Positivity	SARS-CoV-2 rtpcr POS Cohort 1 N = 48	SARS-CoV-2 rtpcr NEG Cohort 2 N = 67	Dengue 2019 Cohort 3 N = 30	Controls Cohort 4	*p*-Value (1)	*p*-Value (2)
SARS-CoV-2 NP IgA n (%)	27/96 (28)		0/30 (0)	9 (0)	<0.0001	
1–8 days	5/29 (18)	22/67 (33)	NA	NA	NA	NA
15–28 days	5/16 (31)	NA	NA	NA	NA	NA
42–62 days	1/3 (33)	NA	NA	NA	NA	NA
65–177 days	0/19 (0)					
SARS-CoV-2 NP IgG n (%)	35/95 (37)		30 (0)	9 (0)	<0.0001	
1–8 days	8/28 (29)	27/67 (40)	NA	NA	NA	NA
15–28 days	6/16 (37)	NA	NA	NA	NA	NA
42–62 days	3/3 (100)	NA	NA	NA	NA	NA
65–177 days	7/19 (37)					
DENV IgM positive n, %	3/36 (8)	2/67 (3)	13/30 (43)	0 (0)	0.25	0.67
DENV IgG positive n, %	33/36 (92)	54/67 (81)	30/30 (100)	4 (44)	0.8	0.11
IgM/IgG ratio mean (95% CI)	0.32 (0.23–0.42)		0.34 (0.24–0.45)	0.6 (0.3–0.9)	0.82 *	
NS1 mean (95% CI)	2 (1.8–2.3)		68 (28–108)	NA	<0.0001 *	

The n = positive reactivity ≥ 10 U/L in ELISA/total number samples and %= percentage positivity. p values from chi square analysis of the positive proportions or * *t*-test of mean values compared between the cohorts 1 and 2 and 3 versus 3 or between 1 versus 2, 1 versus 3.

## Data Availability

Data sharing not applicable. No new data were created or analyzed in this study. Data sharing is not applicable to this article.
